#  Self-assembly and elasticity of hierarchical proteoglycan–hyaluronan brushes†
†Electronic supplementary information (ESI) available: Variations in areal mass density upon SLB and SAv monolayer formation determined by SE (Fig. S1). See DOI: 10.1039/c3sm51213d



**DOI:** 10.1039/c3sm51213d

**Published:** 2013-07-22

**Authors:** Seetharamaiah Attili, Ralf P. Richter

**Affiliations:** a CIC biomaGUNE , Biosurfaces Unit , Paseo Miramon 182 , 20009 San Sebastian , Spain . Email: rrichter@cicbiomagune.es ; Tel: +34 943 0053 29; b Max Planck Institute for Intelligent Systems , Heisenbergstraße 3 , 70569 Stuttgart , Germany; c J. Fourier University , Department of Molecular Chemistry , Laboratory I2BM , 570 Rue de la Chimie , 38041 Grenoble Cedex 9 , France; d University of the Basque Country , Department of Biochemistry and Molecular Biology , Barrio Sarriena s/n , 48940 Leioa , Spain

## Abstract

Spatially confined yet strongly hydrated assemblies made from the proteoglycan aggrecan and the polysaccharide hyaluronan (HA) are major, functionally important components of the pericellular space around chondrocytes, and in cartilage. To better understand, how mechanical properties arise from the supramolecular structure and dynamics of such assemblies, we have studied the effect of aggrecan on the physico-chemical properties of well-defined, planar HA brushes. From interaction studies by quartz crystal microbalance with dissipation monitoring and spectroscopic ellipsometry, and compression studies by combined colloidal probe atomic force/reflection interference contrast microscopy, we find that aggrecan readily intercalates into HA brushes in a reversible manner. Aggrecan induces a drastic swelling of HA brushes, generating self-assembled films of several micrometers in thickness that are highly hydrated (>99%), elastic and very soft. The Young modulus in the linear compression regime is well below 100 Pa, and reaches several kPa at strong compression. The implications of these findings for biological function are discussed.

## Abstract 2

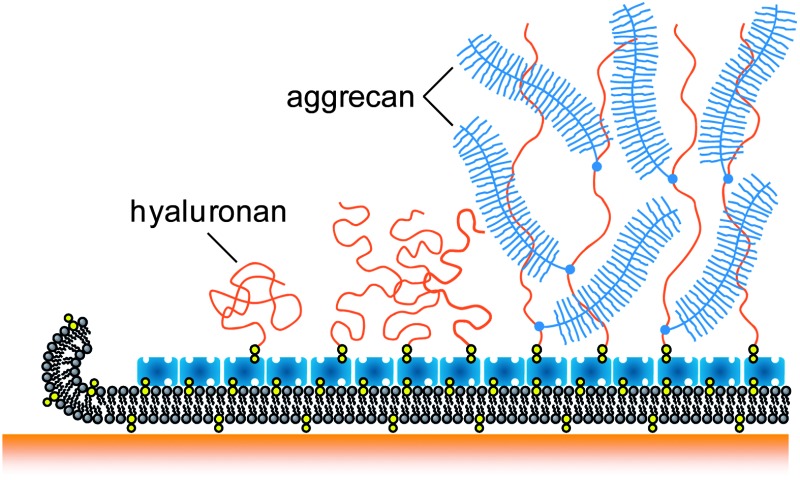
We assemble aggrecan-containing hyaluronan brushes to study how the supramolecular structure and dynamics relate to material properties in hyaluronan-rich pericellular matrices.

## Introduction

Many mammalian cells are surrounded by a very soft and strongly hydrated coat that is rich in the glycosaminoglycan (GAG) hyaluronan (HA). Such pericellular coats (PCCs) can be between 100 nm and many micrometers thick. The PCC influences vital cellular processes such as cell adhesion,^1,2^ proliferation^3–5^ and motility,^6,7^ and plays important roles in numerous physiological and pathological processes, such as inflammation,^8^ embryogenesis,^9^ tumor development,^10–13^ osteoarthritis and atherosclerosis.^14^

HA is a linear polymer of disaccharides that is negatively charged under physiological conditions. Each disaccharide has a length of 1 nm,^15^ and is made of glucuronic acid and *N*-acetylglucosamine. *In vivo*, hyaluronan is expressed by HA synthases at the cell membrane and extruded into the extracellular space. These HA molecules have a molecular mass of typically a few million Daltons,^9^ and a contour length of several micrometers. They can stay attached to the synthases and/or bind to other HA receptors at the cell surface, such as CD44,^16^ to form PCCs, or be released into the extracellular space.

PCCs do also contain hyaladherins, *i.e.*proteins that bind specifically to HA. Depending on their nature and abundance, hyaladherins may cross-link, collapse, stiffen or swell individual HA chains, and thereby modify the morphology and physico-chemical properties of HA assemblies.^8,9,13,14,17–20^ Perhaps the most remarkable hyaladherin in terms of its molecular design is aggrecan. Aggrecan is a large bottle-brush shaped proteoglycan.^21–23^ The contour length of the core protein is typically 350 nm and many negatively charged GAG side chains (chondroitin sulfate and keratan sulfate) extend about 30 nm from the core protein.^22^ Aggrecan and HA can assemble into large multimolecular complexes,^24^ which are an essential component of cartilage, and of the pericellular space around chondrocytes which are interspersed in cartilage.

Numerous biological functions have been related to the mechanical properties of PCCs.^8,25–27^ Over recent years, methods have been emerging to assess the mechanics of native PCCs, *e.g.* for cells in culture^28–32^ or for the endothelial cell surface in blood capillaries.^25^ Such studies provide meaningful information about variations in PCC properties between cell types (*e.g.* in disease), as a function of the cell or environmental stimuli, or across the pericellular space. It remains difficult, however, to understand the rules of PCC assembly and how mechanical and other physico-chemical properties are connected to the PCCs supramolecular structure and dynamics. This is so because the composition of the PCC around living cells is difficult to quantify and the supramolecular organization of PCCs is difficult to image at high resolution: due to the strong hydration, imaging contrast is very low and the assemblies are destroyed upon drying.

Tailor-made model systems that contain a selected subset of the PCC's components and that recapitulate the two-dimensional confinement and the self-assembly properties of the PCC can be used to address this challenge.^16,20,33,34^ Here, we make use of well-defined planar films of end-grafted HA, so-called HA brushes^33,35^ to study the effect of aggrecan on the morphology and mechanical properties of confined HA assemblies. Due to the confinement of the HA assembly to a solid support, the physico-chemical properties of the HA films as a function of external cues become accessible to investigation by surface-sensitive biophysical techniques.^33–35^ We employ quartz crystal microbalance with dissipation monitoring (QCM-D) and spectroscopic ellipsometry (SE) to monitor film formation and to quantify the binding kinetics of aggrecan. The mechanical properties of composite HA–aggrecan films are then characterized by combined colloidal-probe atomic force/reflection interference contrast microscopy (colloidal-probe AFM/RICM) and compared with HA brushes alone.

We demonstrate that the intercalation of aggrecan, even at relatively low densities, induces morphological changes of remarkable magnitude in HA brushes, leading to the formation of extremely thick, soft and hydrated films. Implications for the formation and properties of HA-rich peri- and extracellular matrices are discussed.

## Results

###  Assembly of aggrecan-containing HA brushes

**Fig. 1:**
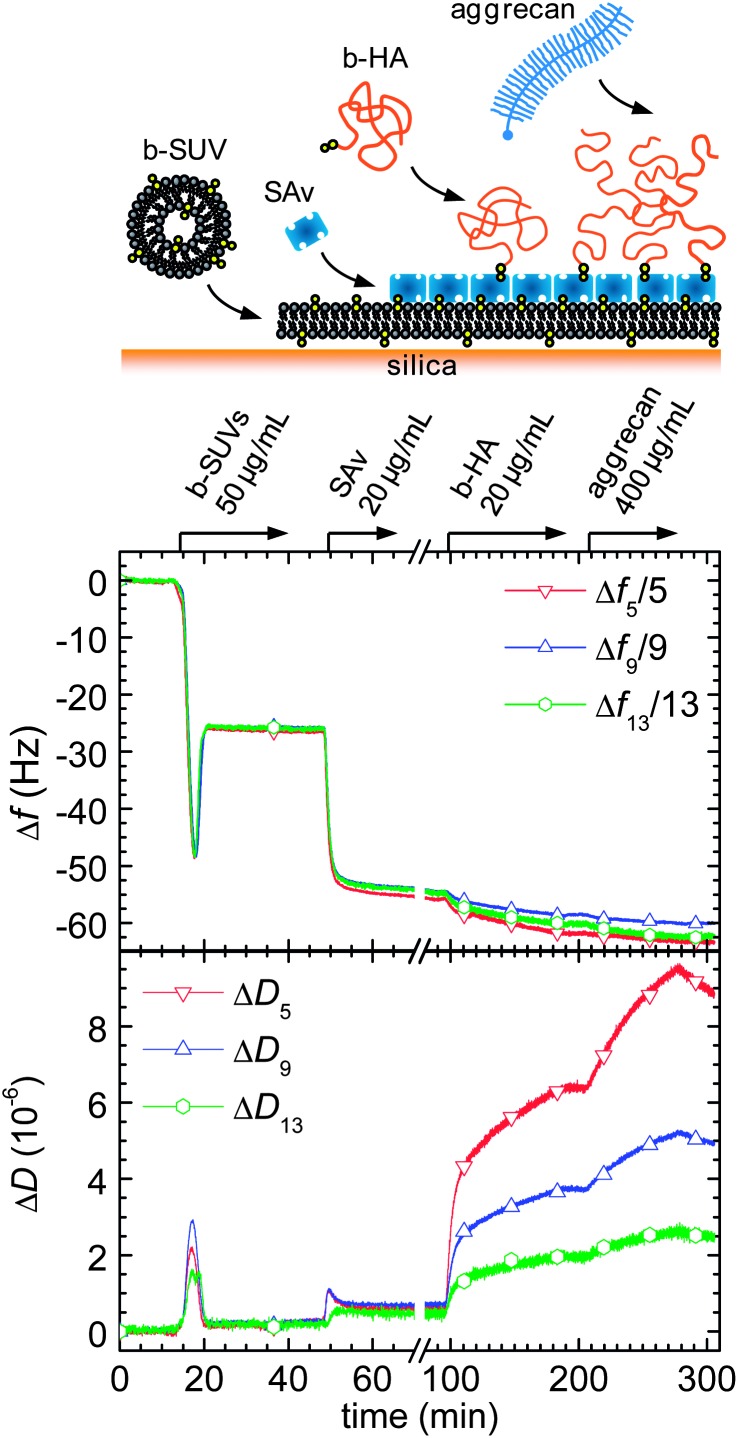
Assembly of an aggrecan-containing HA brush monitored by QCM-D. Shifts in frequency (Δ*f*) and dissipation (Δ*D*) at selected overtones (*n* = 5, 9, 13) are shown. The start and duration of incubations with samples are indicated by arrows on top of the panels; during remaining times, the sensor surface was exposed to pure buffer solution; assembly steps are also schematically illustrated (top). The QCM-D responses for the first two incubation steps are consistent with the formation of a supported lipid bilayer of good quality and the formation of a dense streptavidin monolayer, respectively. The large dissipation shifts, as well as the spread in the overtones, upon incubation with b-HA are consistent with the formation of a soft and highly solvated brush. The soft and highly solvated nature of the film is retained upon reversible binding of aggrecan.

 Quartz crystal microbalance with dissipation monitoring (QCM-D) was used to ascertain the correct assembly of HA brushes and for an initial characterization of aggrecan binding (Fig. 1). First, the silica-coated QCM-D sensor was functionalized with a streptavidin (SAv)-coated supported lipid bilayer (SLB). The two-phase response upon exposure of 50 μg mL^–1^ biotinylated small unilamellar vesicles (b-SUVs) to the sensor surface (Fig. 1, 15 to 30 min) is characteristic for the formation of a SLB by a process in which the vesicles first adsorb intact and then rupture and spread.^36,37^ The equilibrium frequency and dissipation shifts were within the limits of Δ*f* = –25 ± 1 Hz and Δ*D* < 0.3 × 10^–6^, respectively, consistent with the formation of an SLB of good quality.^38^ A further decrease in frequency (by –28 Hz; Fig. 1, 50 to 70 min), with a minor increase in dissipation (about 0.3 × 10^–6^) occurred upon subsequent exposure of 20 μg mL^–1^ SAv. The pattern of frequency and dissipation shifts agrees very well with earlier reports for the formation of a dense monolayer of SAv in which the SAv molecules are anchored stably and with a well-defined orientation to the biotinylated SLB.^35,39^

Grafting of HA was accomplished by incubating the SAv monolayer for 90 min (Fig. 1, 95 to 185 min) with 20 μg mL^–1^ of HA displaying a biotin moiety at its reducing end (b-HA; 0.84 MDa). This step resulted in detectable but small changes in frequency and large shifts in dissipation. The pronounced spreading of the dissipation responses as a function of overtone, the elevated changes in the dissipation shifts and the small changes in frequency indicate the formation of a very soft and hydrated film, as expected for a HA brush and reported earlier.^35^ No further changes were observed after rinsing with buffer solution (Fig. 1, 185 to 205 min), *i.e.* HA was stably grafted.

When 400 μg mL^–1^ aggrecan were added to the HA brush (Fig. 1, 205 to 275 min), no major changes in frequency but a large increase in dissipation occurred, indicating interaction. The similarity in response compared with the HA brush formation provides evidence that the film remained very soft and hydrated even after proteoglycan incorporation. Partial reversal of the responses upon rinsing with buffer solution (Fig. 1, after 275 min) indicated that binding of aggrecan to the HA film is reversible.

### Quantification of adsorbed amounts

*In situ* spectroscopic ellipsometry (SE) was employed to quantify the areal mass density *Γ* of lipids, SAv, HA and aggrecan, and to obtain a first estimate of the film thickness *L* throughout film formation. Here, a silicon wafer served as a substrate which exposes a thin film of silicon oxide on its surface, similar to the QCM-D sensor. The sample incubation protocol for the SE measurements was also kept similar to the QCM-D measurements.

To obtain the film properties (Table 1, Fig. 1 and Fig. S1†) from the measured ellipsometric parameters, SE data were fitted with a model of multiple laterally homogenous layers. The lipid film with or without SAv was considered a single biomolecular layer (index SLB/SAv), and treated as a homogeneous and transparent Cauchy medium, where the first Cauchy parameter *A*_SLB/SAv_ is a measure of the refractive index. The added areal mass densities after incubation with lipids and with SAv were 380 ng cm^–2^ and 280 ng cm^–2^ (Fig. S1†), in agreement with expectations for an SLB,^40^ and for a dense SAv monolayer,^41^ respectively. Both layers were also completely stable to rinsing in buffer solution (Fig. S1†) as already seen by QCM-D (Fig. 1).

**Table 1:** Areal mass densities, thickness values and optical properties determined by SE at the end of each sample incubation step

Biomolecular layer	Δ*A* ^*a*^	*L* (nm)	*Γ* (ng cm^–2^)
SLB	0.116	5.6	380
SLB + SAv	0.109	10.6	380 + 280
HA	2.6 × 10^–4^	505	58
HA + aggrecan	8.6 × 10^–4^	1500	58 + 520

^*a*^Difference in the first Cauchy parameter *A* between the respective layer and buffer, equivalent to the refractive index difference. The values for HA with and without aggrecan refer to the optical properties at the base of the layer, assuming a parabolic refractive index profile.

**Fig. 2:**
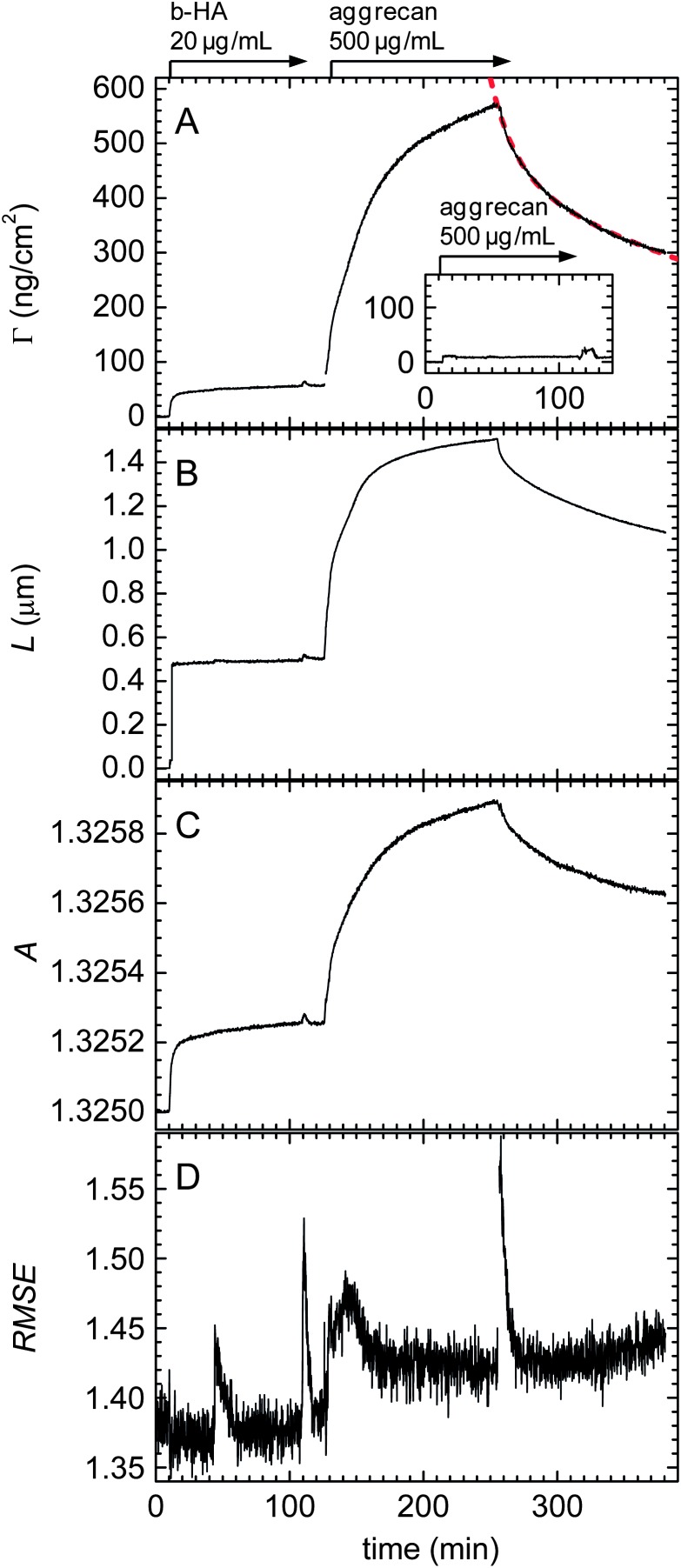
Areal mass density *Γ* (A), film thickness *L* (B) and first Cauchy parameter *A* (C) during HA brush formation and aggrecan loading, as measured by SE. The start and duration of incubations with samples are indicated by arrows on top of the panels; during remaining times, the surface was exposed to pure buffer solution. The red dashed line in A is a double exponential fit (eqn (1)). The inset in A shows the response for the exposure of aggrecan to a SAv monolayer in the absence of HA. (D) Root mean square errors (RMSE) for the determination of *L* and *A* through numerical fitting of SE data.

The HA film with or without aggrecan was considered a separate, second biomolecular layer (index HA). We know from a previous study^33^ that pure HA brushes exhibit an approximately parabolic density profile at physiological ionic strength, and therefore treated this layer as a transparent Cauchy medium with a parabolic refractive index profile. Throughout the fitting, the root mean square error (RMSE, Fig. 2D) varied only moderately and remained close to 1, *i.e.* the model indeed reproduced the data well.

Representative data derived from an SE measurement for the formation of an HA brush through grafting from a solution of 20 μg mL^–1^ b-HA (1.08 MDa) for 100 min is shown in Fig. 2 (10 to 110 min). The final brush thickness was 505 ± 18 nm, where the error corresponds to the confidence interval extracted with the fitting software. Several observations suggest that the error on the thickness is considerably larger, if one takes systematic measurement errors into account. First, *L*_HA_ was sensitive to the choice of *L*_SLB/SAv_: the value of *L*_HA_ = 505 nm was obtained when simultaneously fitting *L*_SLB/SAv_; when *L*_SLB/SAv_ was instead fixed to a value that deviated by as little as 0.4 nm (corresponding to 22 ng cm^–2^ of SAv, or the drift that we observed over a time scale of 1 h, see Fig. S1†) from the best fit, a thickness of 451 nm was obtained. Second, three different experiments under similar experimental conditions yielded brush thickness values between 480 and 600 nm (data not shown). In an earlier study,^33^ we had determined the thickness of an HA brush made with similar experimental conditions from a fit to experimental compression force curves with a model based on self-consistent mean-field theory that predicts a parabolic profile. The value with that approach was 619 nm, and the results are in reasonable agreement if one considers the relatively large error associated with the determination of *L*_HA_ by ellipsometry.

The areal HA mass density after rinsing was 58 ng cm^–2^. This value was robust, *i.e.* it varied only within a few percent between measurements and for the different fitting routines described above. Based on a molecular mass of 1.08 MDa, the surface area per chain *s* ^2^ can be calculated as 3.2 × 10^3^ nm^2^, corresponding to *s* = 57 nm. This value is in excellent agreement with the value of 57 nm obtained previously through the fitting of force curves.^33^

In the course of a subsequent incubation with 500 μg mL^–1^ aggrecan for 2 h (Fig. 2, 135 to 255 min), the film thickness increased to approximately 1500 nm, *i.e.* the proteoglycan induced a drastic swelling of the HA film. The thickness increase compared to the pure HA brush was almost 1 μm, *i.e.* much larger than the contour length of aggrecan (350 nm), whereas the total film thickness remained below the contour length of HA (2.9 μm). Aggrecan is not known to form supramolecular complexes in the absence of HA and previous studies by others have provided evidence that it does not assemble into multilayers when immobilized on a planar support.^42,43^ Therefore, we propose that the thickness increase must be the result of HA chain stretching, as a consequence of the penetration and binding of aggrecan molecules into the HA brush.

The initial binding of aggrecan was slow when compared, for example, with the binding of SAv to an SLB (Fig. S1†). Even after two hours of incubation, aggrecan binding did not reach equilibrium. Presumably, as more aggrecan molecules bound to the HA brush, steric hindrance increasingly limited the access of aggrecan to the HA film and thereby slowed the adsorption. The amount of bound aggrecan at the end of incubation was approximately 520 ng cm^–2^ (Fig. 2A). Based on an aggrecan molecular mass of ∼2.75 MDa (ref. 44) and the surface density of HA, this would correspond to an average of about 3.5 aggrecan molecules per HA chain.

The SE data confirmed the previous observation by QCM-D that aggrecan binding is reversible. The film thickness gradually decreased upon desorption of aggrecan, to approximately 1.1 μm after 2 h of rinsing (Fig. 2B). At this time, approximately 50% of the aggrecan had desorbed from the film (Fig. 2A). Aggrecan binds to HA through its N-terminal G1 domain, and this interaction is known to be relatively weak, *i.e.* dissociation constants of 0.23 μM have been reported.^45^

To estimate the dissociation rate of aggrecan from HA brushes, we fitted the areal mass density data (Fig. 2A, 255 to 390 min) after rinsing with a double exponential1
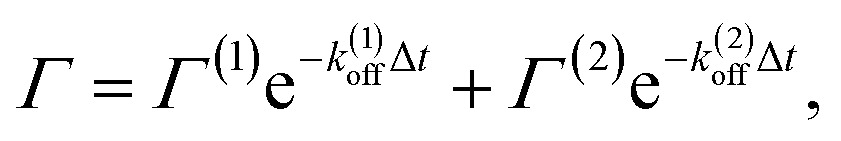
where *Γ* is the areal mass density of aggrecan, Δ*t* is the rinsing time, *k*(1)off and *k*(2)off are desorption rate constants and *Γ* ^(1)^ and *Γ* ^(2)^ the associated areal mass densities. The quality of the fit (red dashed line in Fig. 2A) was good. The first dissociation rate constant was 9.0 × 10^–4^ s^–1^. The second dissociation rate constant was 6.2 × 10^–5^ s^–1^, that is, one order of magnitude smaller. The associated areal mass densities were 130 and 390 ng cm^–2^, respectively, *i.e.* binding was dominated by *k*(2)off. In the simplest interpretation, one may associate the two different rate constants with two discrete unbinding processes. The data, however, are also consistent with the presence of a spectrum of unbinding processes with many different dissociation rates. The latter interpretation appears plausible if one considers that the aggrecan molecules are likely to intercalate into the HA brush at various depths, and therefore will take different times to make their way out of the film.

To confirm that aggrecan binds specifically to HA, interaction with a SAv monolayer in the absence of HA was tested (Fig. 2A, inset). Only a minor increase of less than 10 ng cm^–2^ in the areal mass density was noticed which is negligible compared to the response on HA brushes.

### Impact of aggrecan on HA brush compression

The impact of aggrecan on the mechanical response of HA brushes was examined with a setup that combines colloidal probe atomic force microscopy (AFM) and reflection interference contrast microscopy (RICM) to measure film indentation.^46^ With this method, indentation forces *F* can be determined as a function of the absolute distance *d* between the colloidal probe and the substrate on which the soft film is deposited. From *d* and *L*, the strain *σ* = 1 – *d*/*L* can readily be determined, a parameter which, for continuous films, is usually not directly accessible with AFM alone.

Sample concentrations, HA molecular mass and incubation times for HA brush formation prior to AFM/RICM measurements were kept identical to the SE measurements. RICM requires transparent substrates, and a glass cover slip was therefore used which promotes SLB formation in a way similar to silicon oxide.^37^ To enhance proteoglycan loading, the HA brush was now exposed to a bulk solution of 1 mg mL^–1^ aggrecan for 8 h. Unbound aggrecan was then removed, and force curves were taken. The time that elapsed between rinsing in buffer and acquisition of force curves was a few hours. We do not know the exact amount of aggrecan in the HA film under these conditions. However, based on the ellipsometric data of the adsorption and desorption kinetics at half the aggrecan concentration in the bulk solution (Fig. 2A), we estimate that the aggrecan content in the film corresponded to a few aggrecan molecules per HA chain.

**Fig. 3:**
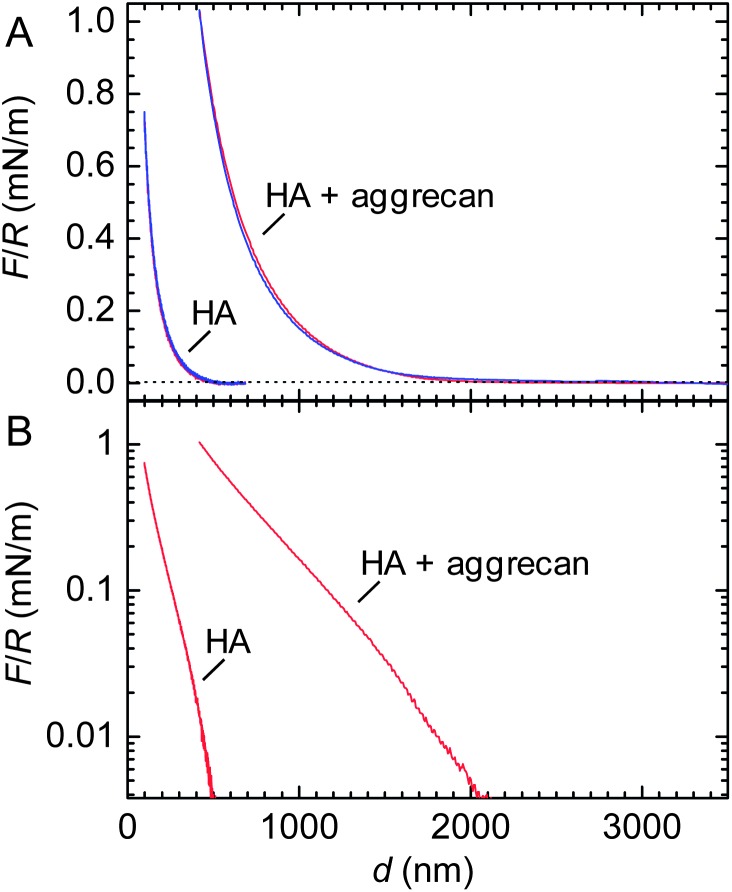
Compressive response of composite HA–aggrecan films. (A) Normalized force (*F*/*R*) *vs.* distance (*d*) for the compression of a film of end-grafted HA, alone and with aggrecan, as indicated (approach – red line, retract – blue line). The dotted line indicates the force threshold of 50 pN that was used to estimate the onset of repulsion and film thickness. (B) Approach data from (A) in a log-linear presentation. Only data above the force threshold are presented. Data for HA alone were taken from Fig. 6 in ref. 46.

Representative curves of the force *F* (normalized by the probe radius *R*) *vs.* distance *d* are shown in Fig. 3. The interactions between the colloidal probe and the HA film were purely repulsive, irrespective of the absence or presence of aggrecan, indicating that biomolecular adhesion to the polystyrene probe did not affect the force curves appreciably. We note that the diameter of the employed colloidal probes, about 25 μm, exceeded the indentation depths by at least one order of magnitude. Therefore, the measured forces reflect the response of the films to compression (rather than penetration^32^). To check reproducibility, force curves were acquired repeatedly at the same spot and subsequently at different positions on the same surface. All curves were very similar, with differences not exceeding those shown between approach and retract curves in Fig. 3A.

Interestingly, approach and retract curves superposed well, indicating that the compression was predominantly elastic. This means that HA and aggrecan in the film can rearrange rapidly, within seconds, and readily regain their original conformation if external stress is released. At the same time, concomitant liquid flow in and out of the compressed area occurs with relative ease, without significant dissipative losses.

In the presence of aggrecan, repulsive forces started to exceed a threshold force of 50 pN (*i.e.* slightly above the noise level) at a distance of *L* = 2.05 ± 0.03 μm, approximately 4 times larger than on pure HA films. This corroborates our finding by SE, that aggrecan induces a remarkable swelling of HA brushes. At any given probe–substrate separation, the resistance of the film to compression was enhanced by aggrecan. For example, at *d* = 420 nm (*i.e.* the point of closest approach for the aggrecan-loaded film), the compression force in the presence of aggrecan was more than 50-fold higher than for HA alone. Despite the large changes in the magnitude of the forces, aggrecan had no appreciable effect on the shape of the force curve: the log-linear representation of *F*/*R vs. d* (Fig. 3B) reveals a roughly exponential dependence in the absence as well as in the presence of aggrecan.

### Young's modulus of composite HA–aggrecan films

To obtain the Young's modulus *E* at low compression (*i.e.* in the linear elastic regime) we fitted the force curves close to the onset of compression with

2 *F*/*R* = π*LE*(1 – *d*/*L*)^2^.

The formula is derived from *E* = *P*/*σ* with the pressure (or stress) *P*: the compression energy (per unit surface area) between two co-planar surfaces can be derived from the compression forces in a sphere/plane geometry using Derjaguin's approximation as *W* = *F*(*d*)/2π*R*;^47^ pressure is *P* = d*W*/d*d*; in the linear elastic regime, *E* is constant, and the fitting formula is obtained by integrating both sides of *Eσ* = *P* with respect to *d*, with the boundary condition *F*(*L*) = 0.

**Fig. 4:**
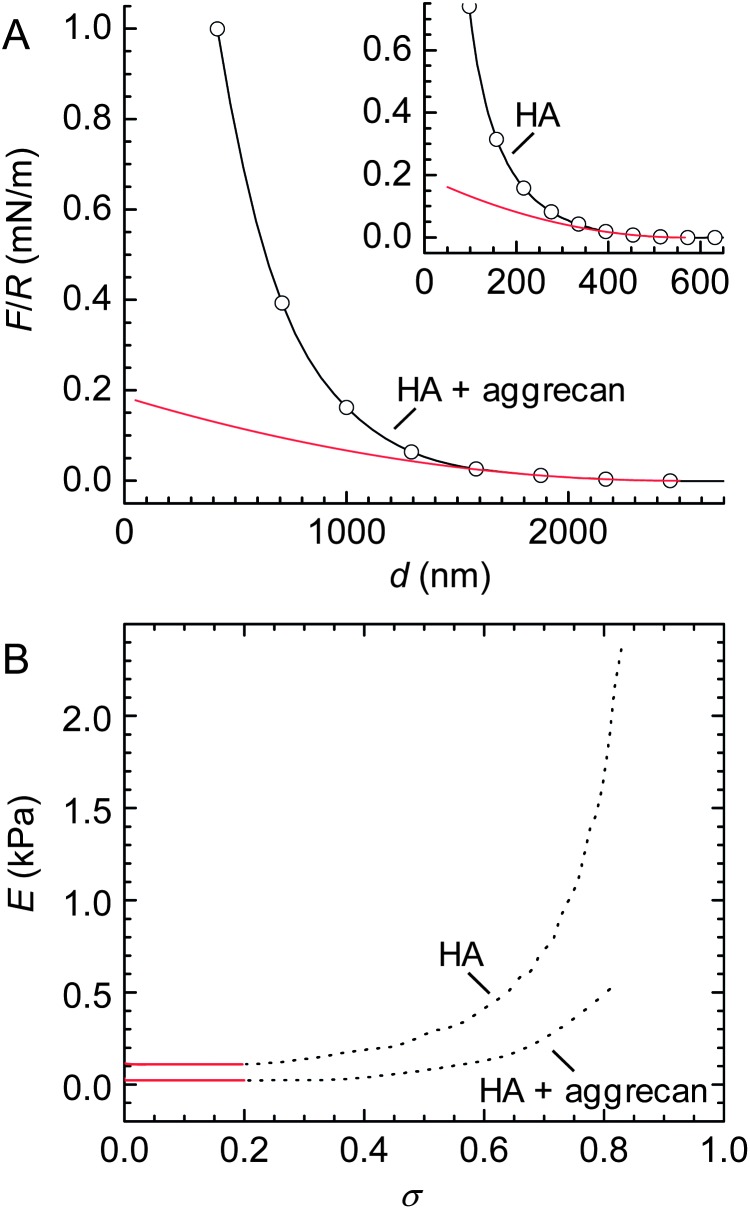
Elasticity of composite HA–aggrecan films: (A) an extrapolated fit with eqn (2) (red line) to a compression force profile (black line with circles) on an aggrecan-loaded HA film (data from Fig. 3). The fit over the range 0 < *σ* < 0.2 provided *L* = 2.50 μm and *E* = 24 Pa. The inset shows the same fit for pure HA brushes for comparison, providing *L* = 0.57 μm and *E* = 108 Pa. (B) Young's modulus *E vs.* strain *σ* for pure HA and composite HA–aggrecan films derived from the approach curves in Fig. 3. Values for *σ* < 0.2 (red solid lines) were taken from the fit to eqn (2); values for *σ* ≥ 0.2 were obtained through the fit to eqn (3) (black dotted lines).

Fig. 4A shows a (extrapolated) fit to the approach curve on a composite HA–aggrecan film. Data up to 20% compression could be fitted well, giving *E* = 24 Pa and *L* = 2.50 μm. A similar fit for a pure HA film gave *E* = 108 Pa and *L* = 0.57 μm (inset).§
§The reader may note that the thickness values obtained through the force threshold of 50 pN were slightly smaller than the values obtained through fitting with eqn (2). For films that exhibit a density gradient along the surface normal, the thickness will depend on the method by which it is measured. Therefore, only an approximate comparison is warranted. At strains above 0.2, the fit underestimated the experimental data, indicating that the elastic response becomes nonlinear. In this regime, stress can be determined from the derivative of the force *vs.* distance curves through^46^

3 *P* = d*W*/d*d* = (2π)^–1^d(*F*/*R*)/d*d*.

The Young's modulus as a function of strain is shown in Fig. 4B. Comparison with pure HA brushes shows that the intercalation of aggrecan makes the HA films somewhat softer: the Young's modulus decreases by 3 to 4 fold at any given strain.

## Discussion

Using an ensemble of surface-sensitive analytical techniques, we have studied the self-assembly and the mechanical properties of composite aggrecan–HA films. Complexes of aggrecan and HA are a major component of chondrocyte PCCs and cartilage. A salient feature that the *in vitro* reconstituted model system shares with these *in vivo* materials is that the complexes are spatially confined: in the model system and in chondrocyte PCCs, two-dimensional confinement arises from the attachment of HA to a planar surface and a cell membrane, respectively; in cartilage, a fibrous collagen network with a rather large mesh size of typically several 100 nm (ref. 48) limits the space available to aggrecan–HA complexes. In contrast to the *in vivo*matrix, the *in vitro* model films are well defined with regard to their composition, morphology and the imposed confinement (end-grafting of HA).

###  Self-assembly kinetics and morphology of aggrecan–HA matrices

**Fig. 5:**
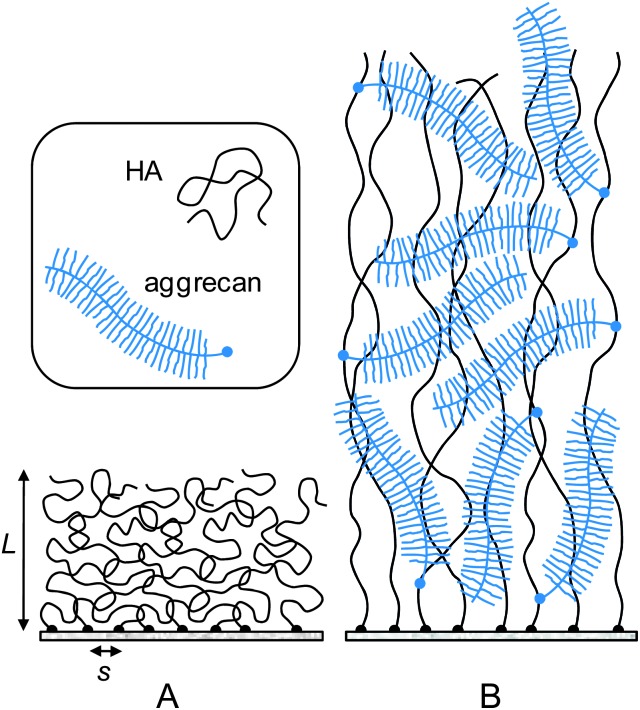
Putative morphology of HA brushes, alone (A) and with aggrecan (B). The cartoon is based on our data and the known molecular interaction between aggrecan and HA. The film thickness, the contour lengths of HA, aggrecan and its GAG side chains are drawn approximately to scale. The diameter of the polymer chains, the grafting distance of HA and aggrecan's GAG chains are enlarged for illustrative purposes.

The drastic swelling of HA films upon incorporation of aggrecan, by several times the original HA brush thickness, is likely to be a direct consequence of two-dimensional confinement. Based on our results, and the known molecular interactions, we propose that the change in morphology is driven by the intercalation of the bulky proteoglycan into the HA brush, as shown schematically in Fig. 5: multiple aggrecan molecules attach *via* their G1 domains to a given HA chain^24^ and a combination of electrostatic repulsion (of the negatively charged GAGs) and volume exclusion (of GAGs and peptides) drives stretching of the HA chains. Although the exact distribution of aggrecan throughout the HA film remains unknown, the result of this self-organization process is a hierarchical proteoglycanassembly, with bottle brushes (aggrecan) being attached to a planar brush (HA).

Such a conformation has been proposed in the past for pericellular coats around chondrocytes.^17,49,50^ Our *in vitro* reconstituted system demonstrates that the combination of grafted HA and aggrecan is indeed sufficient to generate such a hierarchical organization under near physiological conditions. It should be stressed that the presentation of HA in the form of an end-grafted film is essential to achieve a self-organization of aggrecan and HA into films that are up to several micrometers thick. Seror *et al.* ^43,51^ recently reported a model system in which HA of similar molecular weight was attached *via* several points along the chain to a surface. In that case, the thickness of the HA film increased only moderately upon addition of aggrecan, and the total film thickness remained on the order of 100 nm and below.

It is remarkable that pronounced film swelling was generated with a relatively low density of aggrecan. The total organic content in the unperturbed composite film, 90% of which is aggrecan, can be estimated to be 4 mg mL^–1^ from the numbers in Table 1. The film's hydration hence amounts to 99.6% by weight. From aggrecan's dimensions (a cylinder of ∼350 nm length and ∼30 nm radius^22^) it can be easily verified that the GAG chains of an isolated proteoglycan molecule pervade a solvent volume corresponding to 99.5% of the molecule's own weight (∼2.75 MDa (ref. 44)). The similarity in hydration indicates that aggrecan molecules fill the HA brush without becoming significantly compressed and without their GAG chains interpenetrating.

To our knowledge, the concentration of aggrecan in chondrocyte PCCs, in cell culture or *in vivo*, is not known. Based on a comparison of the effective mesh sizes (see further below), we suggest that the aggrecan content and the hydration of PCCs is comparable to our model films. In contrast, the aggrecan content and the hydration of cartilage were reported to be about 8% (ref. 52) and between 68 and 85%,^53^ respectively. Clearly, HA–aggrecan complexes in cartilage are much more strongly confined than in our unperturbed films: to reach a comparable aggrecan concentration, our HA films would have to be compressed to about 100 nm.

In principle, the incorporation of aggrecan into HA films may be thermodynamically or kinetically limited. Considering that the affinity between HA and aggrecan is rather weak, one might argue that thermodynamic limitations are important for the self-assembly process and that the cartilage link protein, which is known to stabilize the bond between aggrecan and HA,^54^ should therefore facilitate the formation of denser HA–aggrecan films. The fact that aggrecan binding did not reach equilibrium after two hours of incubation (Fig. 2), however, demonstrates that kinetic effects play an important, perhaps even dominant, role. Most likely, the surface-confined, interpenetrating HA chains (together with already bound proteoglycan) constrain the diffusion of the bulky aggrecan into the HA brush. In the pure HA brush, the correlation length *ξ*_HA_ (a measure for the mesh size) is expected to be similar to the average distance *s* between anchor points on the surface.^55^ This value is 57 nm in our case, indeed comparable to the smallest dimensions of aggrecan.

The HA concentration in our HA films is comparable to concentrations reported *in vivo*, in particular in synovial fluid^56^ and cartilage.^57^ The kinetic limitations that we observe *in vitro* may hence also be relevant *in vivo*. *In vivo*, aggrecan (with link protein) is secreted by chondrocytes while HA is produced directly at the chondrocyte cell surface. The formation of aggrecan–HA complexes, therefore, is an extracellular process. In this context, it is remarkable that aggrecan–HA complexes in cartilage can be very dense: occupancies as high as one aggrecan per 20 nm of a HA chain contour were found for complexes comprising HA and aggrecan (with link protein) that were isolated from cartilage.^24^ In contrast, with 3.5 aggrecan molecules bound on average per HA chain in our HA films, each aggrecan molecule would have approximately 800 nm of HA contour length at its disposal. One way to assemble the dense complexes observed in cartilage could be through the coordinated delivery of aggrecan and HA at the chondrocyte cell surface. A coordinated regulation of HA and aggrecan content in cartilage has indeed been reported.^58^ Otherwise, sophisticated mechanisms would be required to ‘package’ the secreted aggrecan to enable efficient diffusion and integration into cartilage.

The above-described considerations about the integration of aggrecan should also be pertinent for other hyaladherins. The V0, V1 and V2 isoforms of versican, for example, share the bulky appearance of aggrecan, with a core protein and numerous pendant GAG chains.^59^ Our findings should therefore be of more general relevance for understanding the formation of HA-rich peri- and extracellular matrices. Moreover, we have recently reported that the inflammation-associated protein TSG-6 can cross-link HA films through the formation of HA-induced TSG-6 oligomers, and that cross-linking can induce a decrease in film thickness from 500 to less than 100 nm.^20^ The 5-fold decrease in film thickness with TSG-6 on the one hand, and the 3 to 4-fold increase with aggrecan on the other, illustrate that HA matrices can be highly responsive, *i.e.* the structural range available for the remodeling of HA-rich matrices is very large.

### Compressive mechanics of composite aggrecan–HA films

Controlled compression with colloidal probe AFM/RICM revealed the composite HA–aggrecan films to be remarkably soft yet elastic. The Young's modulus of 24 Pa in the linear elastic regime is on the lower end of elasticity values reported for extracellular matrices in mammals.

Because the film is elastic, we can identify the measured Young's modulus *E* = 24 Pa (Fig. 4A) with the plateau modulus. To a first approximation, the plateau modulus relates to the correlation length of the aggrecan–HA meshwork as *E* ≈ *kT*/*ξ* ^3^, where *kT* = 4.1 × 10^–21^ J is the thermal energy.^60^ This approximation is rather crude, because it neglects the hierarchical organization as well as potential composition gradients in the film, and because the equation is only accurate to within a numerical pre-factor of order unity. The result, *ξ*_HA–aggrecan_ ≈ 55 nm, should therefore be considered an effective value. The correlation length of the composite film is comparable to the correlation length of the pure HA film (*ξ*_HA_ ≈ *s* = 57 nm), suggesting that aggrecan induces film swelling without drastically affecting the effective mesh size.

McLane *et al.* ^32^ have recently mapped the mesh size in PCCs of cultured rat chondrocyte joint cells using a quantitative particle exclusion assay. The authors found the mesh size to increase with the distance from the cell surface, from below 100 nm to about 500 nm. The agreement in magnitude with the effective correlation length in our composite films is reasonable, providing further indication that the reconstituted films reproduce key features of the native PCC.

The mechanical properties of pure aggrecan assemblies have previously been investigated by Dean *et al.* ^61,62^ In their case, aggrecan was covalently grafted *via* one end of its core protein to a planar support.^42^ The resulting assembly is similar to our films in that a hierarchy of brushes is formed, although the type of organization – a planar brush of bottle brushes – is different from our bottle brushes in a planar brush. Interestingly, the shape of the force–distance curves for pure aggrecan brushes is similar to what we found for pure HA and composite HA–aggrecan brushes (Fig. 3B): re-plotting Fig. 8 in ref. 61 on a log-linear scale, one obtains a straight line, *i.e.* force depends approximately exponentially on distance. The elasticity of pure aggrecan brushes at a given strain was more than 2 orders of magnitude higher.^61^ Most likely, this difference is the result of a much higher packing density of the proteoglycan in the pure aggrecan brushes.^61^

Moreover, the force response for our HA–aggrecan brushes is very similar in shape and magnitude to what Sokolov *et al.* ^28^ measured by colloidal probe AFM on epithelial cells. The authors attributed this response to a combination of a polysaccharide-rich coat on the cell surface and cell membrane corrugations. The frequent occurrence of exponential force profiles for polysaccharide-rich films is intriguing, and deserves further investigation.

HA–aggrecan assemblies are a key component of cartilage. At 90% compression, the pressure in our composite HA–aggrecan brushes would be ∼0.5 kPa. This is up to four orders of magnitude weaker than the macroscopic pressure that can be attained in joint cartilage (up to 20 MPa),^63,64^ *i.e.* the HA–aggrecan film would become extremely compressed when exposed directly to the macroscopic pressure in joints. This implies that additional mechanisms must exist to absorb the compressive load. First, a coarse-meshed fibrous collagen network supplements the HA–proteoglycan meshwork in cartilage.^48^ The microscale stiffness of the collagen network has been estimated to be on the order of 1 MPa,^65,66^ *i.e.* collagen could indeed carry most of the compressive load, and attenuate compression of the HA–proteoglycan meshwork.^66^ Stolz *et al.* ^65,66^ estimated a nanoscale stiffness of a few 10 kPa for the proteoglycan meshwork between the collagen fibres. This number is still more than an order of magnitude larger than the linear elastic modulus in our films; extrapolation of the curves in Fig. 4B, however, suggest that a film that is pre-compressed to about 10% of its original thickness would have the appropriate elasticity. Second, our reconstituted HA–aggrecan films lack the cartilage link protein, that is known to stabilize the bond between aggrecan and HA.^54^ Future studies that incorporate the link protein into the model films should provide insight as to how strongly an increase in film stability can enhance the compressive resistance of composite HA–aggrecan films. Third, peak pressures of 20 MPa in cartilage typically occur transiently under high strain rates. Under such non-equilibrium conditions, the resistance to pressure can increase drastically due to delayed drainage of water and ions.^48^ Future studies at higher loading rates should provide interesting insight into the dynamics of fluid retention in HA films.

## Conclusions

We have developed an *in vitro* supported lipid bilayer based model system to study the formation and mechanical properties of HA–aggrecan composite matrices. The intercalation of aggrecan into HA brushes is slow. It ultimately leads to the formation of an elastic film that is hierarchically organized, as bottle brushes in a planar brush, remarkably thick and extremely soft and hydrated. The generated data represent a valuable reference for quantitative studies of HA-rich pericellular coats, and help to rationalize how the supramolecular structure and dynamics relate to material properties in hyaluronan-rich matrices.

## Experimental

### Preparation of sample solutions

A working buffer solution of 10 mM HEPES, pH 7.4 with 150 mM NaCl and 3 mM NaN_3_ in ultrapure water was used in all experiments. 2 mM CaCl_2_ was added for the formation of SLBs.

To prepare small unilamellar vesicles (SUVs), lyophilized dioleoylphosphatidylcholine (DOPC) and dioleoylphosphatidylethanolamine–CAP–biotin (DOPE–CAP–biotin) (Avanti Polar Lipids, Alabaster, AL, USA) were first dissolved in chloroform, mixed in a molar ratio of 9 : 1, dried, re-suspended in buffer solution at 2 mg mL^–1^ concentration and homogenized, as described earlier.^67^ SUVs were then obtained by sonication, as described earlier,^68^ and stored at 4 °C.

Lyophilized hyaluronan (HA), biotinylated at its reducing end and with well-defined molecular weights of either 1.08 ± 0.06 or 0.84 ± 0.04 MDa (*i.e.* two different batches of Select-HA B1000; Hyalose, Oklahoma City, OK, USA), was dissolved in ultrapure water at a concentration of 1 mg mL^–1^, and gently shaken for 2 hours. Lyophilized SAv (Sigma) was dissolved in ultrapure water at 1 mg mL^–1^. Aggrecan (Sigma) was dissolved in buffer solution at 2 mg mL^–1^. Polysaccharide and protein stock solutions were aliquoted and stored at –20 °C.

### Preparation of substrates

Silica-coated QCM-D sensors (QSX303, Biolin Scientific, Västra Frölunda, Sweden) were used as substrates in QCM-D experiments. The sensors were cleaned by immersion in a 2% sodium dodecyl sulfate solution for 30 min, thorough rinsing with ultrapure water followed by blow-drying with nitrogen gas.

Silicon wafers with a native oxide layer of about 2 nm (University Wafers, South Boston, MA, USA) were used as substrates in SE experiments. The wafer was cut to pieces of desired size (30 × 10 mm^2^) to fit into the custom-made ellipsometry cuvette. The wafer pieces were rinsed in ultrapure water and blow-dried with nitrogen gas.

Glass cover slips (#1.5, 24 × 24 mm^2^; Menzel-Gläser, Thermo Scientific, Germany) were used as substrates in colloidal probe AFM/RICM measurements. They were cleaned by rubbing with a lint-free tissue paper and immersion in freshly prepared piranha solution (3 : 1 (v/v) mixture of concentrated H_2_SO_4_ and 50% H_2_O_2_) for 1 h, rinsed in ultrapure water and blow-dried with nitrogen gas.

Substrates were stored in sealed petri dishes, and exposed to UV/ozone (UV/Ozone ProCleaner; Bioforce Nanoscience, Ames, IA, USA) for 30 min prior to use.

###  Quartz crystal microbalance with dissipation monitoring (QCM-D)

QCM-D measures changes in resonance frequency, Δ*f*, and dissipation, Δ*D*, of a sensor crystal upon interaction of (soft) matter with its surface. The QCM-D response is sensitive to the mass (including hydrodynamically coupled water) and the mechanical properties of the surface-bound layer.^69^ To a first approximation, a decrease in Δ*f* indicates a mass increase, while high (low) values of Δ*D* indicate a soft (rigid) film.

QCM-D measurements were conducted with a Q-Sense E4 system (Biolin Scientific, Västra Frölunda, Sweden) using flow modules. The system was operated in flow mode with a rate of typically 5–20 μL min^–1^, using a syringe pump (KD Scientific, Holliston, MA, USA), at a working temperature of 23 °C. Δ*f* and Δ*D* data were collected with sub-second time resolution at six overtones (*n* = 3, 5, 7, 9, 11, 13), corresponding to resonance frequencies of ∼15, 25, 35, 45, 55, 65 MHz. Changes in dissipation, Δ*D*, and normalized frequency, Δ*f* = Δ*f*_*n*_/*n*, of selected overtones are presented.

###  Spectroscopic ellipsometry (SE)

 Ellipsometry measures changes in the ellipsometric angles, *Δ* and *Ψ*, of polarized light upon reflection from a planar sample surface. We employed ellipsometry*in situ* at ambient temperature, using a custom-designed open glass cuvette.^68^ Data were acquired with a spectroscopic rotating compensator ellipsometer (M2000V; J. A. Woollam, Lincoln, NE, USA) over a wavelength range of *λ* = 380–1000 nm, at 70° angle of incidence and with a time resolution of 5 s.

### Fluid handling

Before use, the glass cuvette was passivated by immersion in an aqueous solution of 10 mg mL^–1^ bovine serum albumin for 20 min, rinsed with ultrapure water and blow-dried with a stream of nitrogen gas. A silicon wafer substrate was then installed and the cuvette filled with approximately 700 μL buffer solution.

Sample and buffer solutions were injected using a micropipette at desired times. Excess liquid was removed using a syringe needle connected to a peristaltic pump (Ismatec, Glattbrugg, Switzerland). Care was taken to maintain the volume constant during the entire experiment. To adjust for liquid losses through evaporation, which were significant during long incubation processes, water was added periodically. To ensure homogenization of the cuvette content, the cuvette was equipped with a magnetic stirrer at the bottom which was kept running during the injection of samples and for an additional 10 s after injection. Adsorption processes were monitored in still solution. To remove excess sample from the solution phase, the cuvette content was diluted by repeated addition of 2-fold excess of buffer solution and removal of excess liquid until the concentration of the soluble sample, as estimated from the number of dilution steps, was below 10 ng mL^–1^. The stirrer was kept running during the rinsing process.

### Quantitative data evaluation

The refractive index, thickness and areal mass density of the biomolecular films were determined by numerical fitting of the SE data over the accessible wavelength spectrum using the software CompleteEASE (J. A. Woollam). The quality of the fit was assessed by monitoring the root mean square error (RMSE). The interface was modelled as a stack of laterally homogenous layers. The optical properties of the cuvette and the substrate, *i.e.* the silicon wafer with a thin silicon oxide overlayer, were calibrated as described previously.^68^

The semi-infinite bulk solution was treated as a transparent Cauchy medium with a refractive index *n*_sol_(*λ*) = *A*_sol_ + *B*_sol_/(*λ*/μm)^2^. Cauchy parameters for the buffer solution, *A*_buffer_ = 1.325 and *B*_buffer_ = 0.00322, were calculated from tables in the literature.^39,45^

A first biomolecular layer that consisted either of lipids alone (ultimately forming a SLB) or additionally of SAv, was treated as a homogeneous and transparent Cauchy medium with *n*_SLB/SAv_ = *A*_SLB/SAv_ + *B*_SLB/SAv_/(*λ*/μm)^2^. *B*_SLB/SAv_ was set equal to *B*_buffer_. The layer thickness *L*_SLB/SAv_ and the Cauchy parameter *A*_SLB/SAv_ were fitted simultaneously. The film thickness and refractive index were then used to determine the areal mass density using de Feijter's equation^70^
4A
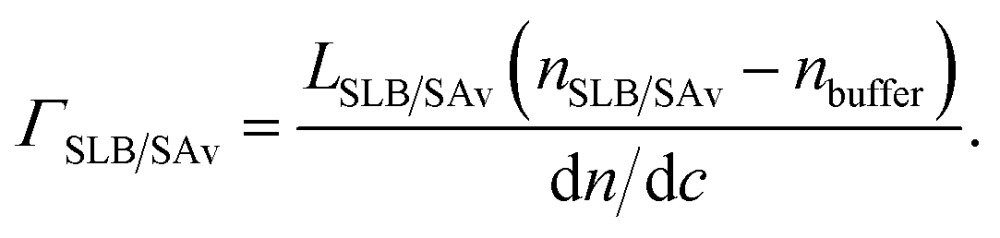



Because *B*_SLB/SAv_ = *B*_buffer_, this equation simplifies to4B
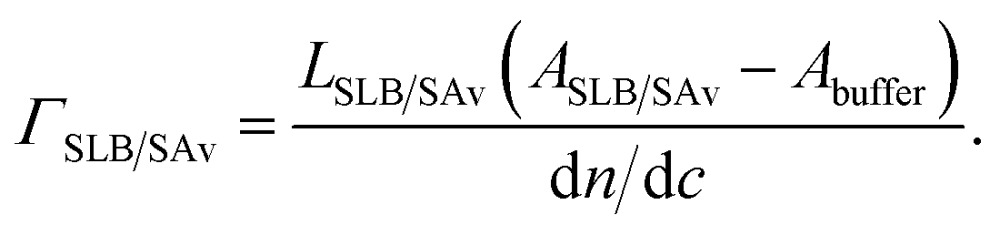



The d*n*/d*c* value represents the increment in the refractive index of the biomolecular film as a function of its concentration *c*. Values of 0.169 cm^3^ g^–1^ for lipids^41^ and 0.18 cm^3^ g^–1^ for SAv^68^ were used.

The HA film was also treated as a transparent Cauchy medium. Because the HA film has a thickness that is comparable to or even larger than the wavelength of the probing light, it is useful to explicitly consider heterogeneities in the direction of the surface normal, *i.e.* the density profile. Pure HA brushes exhibit an approximately parabolic density profile at physiological ionic strength,^33^ *i.e.*

5A *c*_HA_(*z*) = *c*_HA,0_(*L*_HA_^2^ – *z*^2^) for *z* ≤ *L*_HA_,

where *z* is the distance from the anchor points of the HA chains in the direction perpendicular to the surface, and *c*_HA,0_ is the HA concentration close to the anchor points. To a good approximation, Δ*n* = *n*_HA_ – *n*_buffer_ = d*n*/d*c* × *c*_HA_, which gives

5B Δ*n*(*z*) = Δ*n*_HA,0_(*L*_HA_^2^ – *z*^2^) for *z* ≤ *L*_HA_.

To approximate the parabolic refractive index profile, the HA film was treated as a non-linearly graded layer, consisting of 10 slices of equal thickness (a larger number of slices gave identical results). Each slice had a constant refractive index, and the sequence of refractive indices approximated a parabola bounded by *n*_HA,0_ = *A*_HA,0_ + *B*_HA,0_/(*λ*/μm)^2^ at *z* = 0 and *n*_buffer_ at *z* ≥ *L*_HA_. Throughout the buildup of the HA film, *A*_HA,0_ and the layer thickness *L*_HA_ were fitted simultaneously. *B*_HA,0_ was set to be equal to *B*_buffer_. We found that the thickness *L*_SLB/SAv_ exhibited minor drifts and that the results for *L*_HA_ and *A*_HA,0_ depended quite sensitively on the choice of this parameter. Therefore, *L*_SLB/SAv_ was also kept as a fit parameter. All other parameters were fixed to the previously determined values. To obtain areal mass densities from the optical properties of a graded layer, de Fejter's equation can be generalized as6A
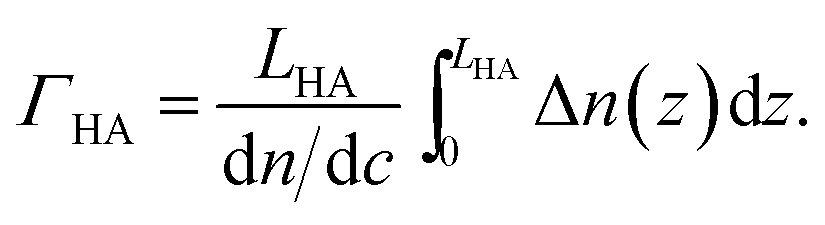



For a parabolic profile, and because *B*_HA,0_ = *B*_buffer_, this gives6B
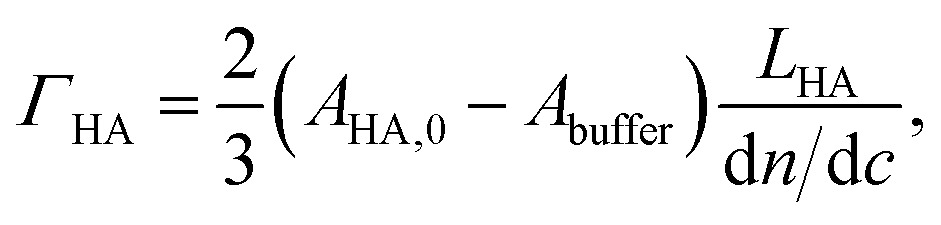
where we used d*n*/d*c* = 0.15 cm^3^ g^–1^ for pure HA films.^71^

The HA film containing aggrecan was also treated as a non-linearly graded layer. Consistent with the treatment of pure HA films, we assumed a parabolic density profile, even though we do not know the exact density profile of this composite film. Aggrecan was exposed to the HA brush at a final concentration of 500 μg mL^–1^. For comparison, the concentration of HA in the strongly hydrated HA brushes is about 1 mg mL^–1^,^46^ *i.e.* of similar order of magnitude. This implies that the increase in the bulk refractive index due to the addition of aggrecan is comparable to the refractive index difference between the HA film and the bulk solution. Therefore, the refractive index of the ambient medium needs to be adjusted in the model. Aggrecan consists of a peptide chain with a molecular mass of ∼250 kDa and many glycosaminoglycan (GAG) chains which total a molecular mass of approximately ∼2.75 MDa.^44^ With De Feijter's equation, we can estimate the refractive index change as Δ*n*_aggrecan_ = [(d*n*/d*c*)_peptide_ × *c*_peptide_ + (d*n*/d*c*)_GAG_ × *c*_GAG_], where *c*_peptide_ and *c*_GAG_ are the peptide and GAG concentrations, respectively, and (d*n*/d*c*)_peptide_ = 0.18 cm^3^ g^–1^ and (d*n*/d*c*)_GAG_ = 0.15 cm^3^ g^–1^. Because the GAG chains dominate, we can simplify to Δ*n*_aggrecan_ ≈ (d*n*/d*c*)_GAG_ × *c*_aggrecan_. For *c* = 500 μg mL^–1^, this gives Δ*n*_aggrecan_ = 0.000075. Whenever aggrecan was present in the ambient solution, *A*_sol_ = *A*_buffer_ + Δ*n*_aggrecan_ = 1.325075 was therefore used when fitting the data instead of *A*_buffer_. Areal aggrecan mass densities were determined through eqn (6B).

###  Colloidal probe AFM/RICM

We used a NanoWizard II with a TAO module (JPK, Berlin, Germany), installed on an inverted optical microscope (Axio Observer D1; Zeiss, Oberkochen, Germany) to combine colloidal probe atomic force microscopy (AFM) with reflection interference contrast microscopy (RICM), as described previously.^46^AFM force curves were acquired in closed-loop mode at an approach speed of *v*_*z*_ = 100 nm s^–1^, and with a maximal load on the order of typically 10 nN. RICM interferographs were acquired simultaneously at two different wavelengths, 438 and 543 nm, using exposure times of typically 100 ms.

Custom-developed algorithms implemented in Matlab were used to quantify the absolute distance between the colloidal probe and the glass substrate from the RICM images. The method, which is described in detail elsewhere,^46^ is based on the analysis of radially averaged intensity profiles with a simple optical model, the parallel plate approximation with incident light parallel to the surface normal.^72^ The correlation of multiple solutions generated by this model enables correction for an imperfectly adjusted focus position and allows for the method to work even if the probe radius is only approximately known. The method provides probe–sample distances with an accuracy of a few nanometers, and the use of two colors extends the range for the unambiguous determination of the distance at closest approach to approximately 1 μm (from about 200 nm for a single color).

The deflection *vs.* piezo displacement curves obtained by AFM were converted into deflection *vs.* relative distance curves using established methods.^73^AFM and RICM data were correlated to adjust for linear thermal drifts in the probe–surface distance and to convert relative distances into absolute distances between the colloidal probe and the planar glass support, as previously described.^46^

### Preparation of colloidal AFM probes

Polystyrene microspheres (Polysciences, Eppelheim, Germany) of 25 ± 3 μm diameter were attached to tipless V-shaped Si_3_N_4_ cantilevers (Veeco Probes, CA, USA) with a nominal spring constant of 0.06 N m^–1^, as previously described.^46^ The real spring constant, determined using the thermal noise method,^74^ was *k* = 0.1 N m^–1^. Prior to use, the cantilevers with a colloidal probe were treated with UV/ozone for not more than 5 min.

### Preparation of the liquid chamber and the HA film

A glass cover slip was attached to a custom-made holder using a two-component glue (Twinsil; Picodent, Wipperfurth, Germany), and the desired biomolecular film was prepared on the glass substrate. The holder was designed to accommodate an AFM liquid cell (SmallVolumeCell; JPK) on the top and a light microscope objective on the bottom. All parts were cleaned and assembled to form an open liquid cell, as described previously.^46^

HA brushes were prepared within a 250 μL droplet of buffer solution on the glass cover slip, using the incubation steps previously established by SE. To remove excess sample after each incubation step, the droplet content was diluted by repeated addition of a twofold excess of buffer and removal of excess liquid until the concentration of the solubilized sample, estimated from the extent of dilution, was below 10 ng mL^–1^. Repeated aspiration and release of the droplet content by a micropipette ensured homogenization at each dilution step. Care was taken to keep the substrate wet at all times.
