# Supplementary material for: Self-assembly and elasticity of hierarchical proteoglycan–hyaluronan brushes
Source: Soft Matter. 2013 Jul 22;9(44):10473–83. doi: 10.1039/c3sm51213d (PMC4080815; doi:10.1039/c3sm51213d)
Supplement: Supplementary file 1 [file SM-009-C3SM51213D-s001.pdf]

Electronic Supplementary Material (ESI) for Soft Matter
This journal is © The Royal Society of Chemistry 2013

Electronic Supplementary Information

Self-assembly and elasticity of hierarchical proteoglycan-hyaluronan brushes

Seetharamaiah Attili,[a,b,d] *and Ralf P. Richter**[a,b,c]

[a] Biosurfaces Unit, CIC biomaGUNE, Paseo Miramon 182, 20009 San Sebastian, Spain

[b] Max Planck Institute for Intelligent Systems, Heisenbergstraße 3, 70569 Stuttgart, Germany

[c] I2BM, Department of Molecular Chemistry, J. Fourier University, 570 Rue de la Chimie, 38041 Grenoble Cedex 9, France

[d] Present address: Randall Division for Cell and Molecular Biophysics, New Hunt's House, Guy's Campus, King's College London, SE1 1UL, UK.

*Corresponding author. Email: rrichter@cicbiomagune.es, phone: +34 943 0053 29, fax: +34 943 0053 15.

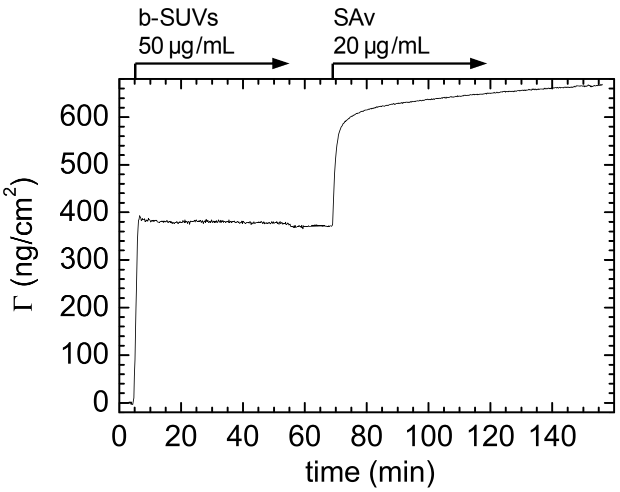

Figure S1. Areal mass density *Γ* during the adsorption of small unilamellar vesicles containing biotinylated lipids (b-SUVs) and streptavidin (SAv), as measured by SE. The start and duration of incubations with samples are indicated by arrows on top of the panels; during remaining times, the silica surface was exposed to pure buffer solution. Areal lipid and protein densities are consistent with a supported lipid bilayer and a dense SAv monolayer, respectively. Close inspection of the binding curve after SAV binding revealed a small but steady increase in *Γ* even after rinsing (~0.36 ng/cm^2/min), the origin of which remains unclear.
